# Lipidomic Predictors of Coronary No-Reflow

**DOI:** 10.3390/metabo13010079

**Published:** 2023-01-03

**Authors:** Arun Surendran, Umar Ismail, Negar Atefi, Ashim K. Bagchi, Pawan K. Singal, Ashish Shah, Michel Aliani, Amir Ravandi

**Affiliations:** 1 Cardiovascular Lipidomics Laboratory, St. Boniface Hospital, Albrechtsen Research Centre, Winnipeg, MB R2H 2A6, Canada; 2 Mass Spectrometry and Proteomics Core Facility, Rajiv Gandhi Centre for Biotechnology, Thiruvananthapuram 695014, Kerala, India; 3 Department of Physiology and Pathophysiology, Rady Faculty of Health Sciences, University of Manitoba, Winnipeg, MB R2H 2A6, Canada; 4 Section of Cardiology, Department of Medicine, Rady Faculty of Health Sciences, University of Manitoba, Winnipeg, MB R3E 0J9, Canada; 5 Department of Internal Medicine, University of Arkansas for Medical Sciences, Little Rock, AR 72204, USA; 6 Faculty of Agricultural and Food Sciences, University of Manitoba, Winnipeg, MB R2H 2A6, Canada

**Keywords:** no-reflow, lipidomics, sphingomyelin, ether-lipids

## Abstract

The ‘no-reflow’ phenomenon (NRP) after primary percutaneous coronary intervention (PCI) is a serious complication among acute ST-segment elevation myocardial infarction (STEMI) patients. Herein, a comprehensive lipidomics approach was used to quantify over 300 distinct molecular species in circulating plasma from 126 patients with STEMI before and after primary PCI. Our analysis showed that three lipid classes: phosphatidylcholine (PC), alkylphosphatidylcholine (PC(O)), and sphingomyelin (SM), were significantly elevated (*p* < 0.05) in no-reflow patients before primary PCI. The levels of individual fatty acids and total fatty acid levels were significantly lower (*p* < 0.05) in no-reflow subjects after PCI. The grouping of patients based on ECG ST-segment resolution (STR) also demonstrated the same trend, confirming the possible role of these differential lipids in the setting of no-reflow. Sphingomyelin species, SM 41:1 and SM 41:2, was invariably positively correlated with corrected TIMI frame count (CTFC) at pre-PCI and post-PCI. The plasma levels of SM 42:1 exhibited an inverse association (*p* < 0.05) consistently with tumor necrosis factor-alpha (TNF-α) at pre-PCI and post-PCI. In conclusion, we identified plasma lipid profiles that distinguish individuals at risk of no-reflow and provided novel insights into how dyslipidemia may contribute to NRP after primary PCI.

## 1. Introduction

Primary percutaneous coronary intervention (PCI) is the choice of revascularization strategy for ST-segment elevation myocardial infarction (STEMI) patients. Nevertheless, myocardial tissue fails to perfuse normally in a small proportion of patients despite the infarct-related artery reopening, a phenomenon known as ‘no-/slow-reflow.’ No-reflow after PCI results in larger infarct size [1] and is influenced by the duration of ischemia and reperfusion [2]. Once it happens, it attenuates the beneficial effect of reperfusion therapy and hurts clinical outcomes. To date, the exact pathophysiological mechanisms underlying no-reflow phenomenon (NRP) remains incomplete.

The no-reflow is a multifactorial process, including atherosclerotic plaque rupture, atheroembolism, ischemic injury, reperfusion injury, and vascular endothelial dysfunction [3,4]. Two recent studies showed that reduced kidney function [5] and the level of glucose at admission [6], are associated with the occurrence of no-reflow. It has been demonstrated that cardiac pericytes constrict coronary capillaries during myocardial ischemia resulting in NRP [7], and reversal of pericyte contraction using pharmacological therapies reduces no-reflow [8]. According to various reports, no-reflow occurs in more than 30% of all patients after PCI [9,10]. Hence, it is important to identify patients at risk of no-reflow prior to PCI.

The most common techniques that can be used to diagnose no-reflow after successful PCI are myocardial contrast echocardiography, TIMI (Thrombolysis in Myocardial Infarction) flow grading, magnetic resonance imaging, myocardial blush and corrected TIMI frame count (CTFC) [11,12]. Previous intravascular imaging studies have demonstrated that the lipid burden of the atherosclerotic plaque is a major discriminator between no-reflow and normal flow in STEMI patients [13]. Furthermore, it has also been reported that the release of plaque contents, including cholesterol crystals, results in embolization of the distal vessels and contributes to no-reflow [14]. This evidence shows that lipid-related disturbances are closely associated with the initiation and progression of no-reflow, and thus, NRP is a suitable candidate for lipidomics studies.

It is now well established that plasma lipids outperform traditional lipid measures (total cholesterol, HDL-C, LDL-C, and total triglycerides) as markers for cardiovascular risk [15,16]. Our previous non-targeted metabolomics analysis in STEMI patients showed that lipids formed the bulk of the altered metabolomic profile in a clinical setting of ischemia/reperfusion (IR) injury [17]. In addition, we showed that selected lipid species could serve as markers of myocardial infarct size. More recently, in lipidomics on the plasma of STEMI patients, we showed that human plasma lipidome rapidly shifts during myocardial ischemia and reperfusion, and specific lipids could predict future cardiovascular events [18]. These studies show that lipid metabolism is perturbed in STEMI patients following PCI. However, no studies have investigated the association between circulating lipids and NRP in STEMI patients after PCI. Here, we aim to do an in-depth lipidomics analysis of human plasma to understand lipids’ role in a no-reflow clinical setting.

## 2. Materials and Methods

### 2.1. Study Population

A total of 126 patients undergoing PCI for STEMI in St. Boniface Hospital between January 2017 and June 2020 were included in the study. Venous blood samples were taken from each patient at the time of presentation (pre-PCI) and 2 h post successful reperfusion (2 h post-PCI). Patients were diagnosed as STEMI based on chest pain onset, confirmation of ST-elevation on 12 lead surface EKG on admission, and documentation of occluded coronary artery with coronary angiography. Blood samples were collected into tubes containing EDTA, and after centrifugation (2500× *g*, 5 min, 4 °C), the extracted plasma samples were immediately stored at −80 °C until needed. The mean time from blood collection to storage at −80 °C was under 20 min.

For each patient, the no-reflow was defined using the corrected TIMI frame count (CTFC) and surface electrocardiography (ECG). The CTFC method, first described by Gibson et al., provides a quantitative assessment of the coronary artery flow [11]. The number of cine frames (at a fluoroscopic rate of 30 frames per second) required for the contrast agent to reach a standardized anatomic landmark in the infarct-related artery was referred to as the CTFC [11]. The cine angiographic examination was initially recorded in the cardiac catheterization laboratory at a rate of 7.5 and 15 frames per second. It was then corrected to 30 frames per second to obtain the CTFC value. Representative normal flow (Supplementary Video S1) and no-reflow (Supplementary Video S2) coronary angiograms has been provided.

### 2.2. Lipid Analysis

Frozen plasma samples were thawed for nearly 20 min. Lipids were extracted using chloroform and methanol as previously described [18,19]. Briefly, 10 μL of plasma was added to 200 μL of chloroform/methanol (2:1, *v*/*v*) along with 30 μL of internal lipid standards (ISTD) in Eppendorf tubes, vortexed for 10 min, and sonicated in a water bath at room temperature (RT) for 30 min. The mixture was allowed to settle for 20 min after which centrifuged at 20,000× *g* for 20 min at RT. The lipid-containing top layer was later transferred to a new Eppendorf tube and dried under a gentle stream of nitrogen gas (∼1 h). The dried samples were then resuspended in 50 μL of water-saturated 1-butanol and sonicated for 10 min, followed by adding 50 μL of 10 mM ammonium formate in methanol. Finally, the extract was spun (10,000× *g* for 10 min at RT), and the supernatant (80 µL) was transferred into glass vials with inserts. The samples were randomized prior to LC/MS analysis. The lipidomic profiling was performed using a Prominence chromatographic system (Shimadzu Corporation, Canby, OR, USA) coupled with an AbSciex 4000 QTRAP triple quadrupole mass spectrometer (AB Sciex, Framingham, MA, USA). The entire LC/MS methodology for this work, including details of ISTD, quality control, and data processing, are described in detail in our previously published work [18].

### 2.3. Cytokine Analysis

Plasma cytokine levels were determined using a custom Meso Scale Discovery kit (MSD, Meso Scale Diagnostics, Rockville, MD, USA) according to the instructions by the manufacturer. The assays were done in 96-well formats, with 10 spots per well. The multiplex panel used a single protocol for the simultaneous quantification of 10 analytes, including interleukins (IL) -1β, 6, 8, 10, tumor necrosis factor-alpha (TNF-α), interferon (IFN)-γ, macrophage inflammatory protein (MIP-1α), interferon-γ-induced protein (IP-10), monocyte chemoattractant protein (MCP-1) and stromal cell-derived factor-1α (SDF-1α). Plates were read with an MSD instrument, and the final values were obtained in pg/mL. Samples with values outside assay range were excluded from the analysis.

### 2.4. Data Normalization

The pre-normalized data was imported into the Mass Profiler Professional (MPP; 12.6) software, and the data were normalized using the ‘percentile shift’ normalization procedure (default 75th percentile). This is a global normalization procedure in which all intensity values in a sample are adjusted. The data was further filtered by omitting the low-intensity values. To this effect, the upper and lower cut-off values for normalized intensity values were set to 100 percent and 20 percent, respectively.

### 2.5. Statistical Analysis

The baseline demographics and clinical characteristics of patients in Table 1 are represented as mean value plus or minus standard deviation (SD) for continuous variables with normal distribution, median (25th, 75th percentiles) for continuous variables with non-normal distribution, and count (%) for categorical variables. The data normality was tested with the Shapiro–Wilk test. Differences in means of these parameters were examined using a student’s *t*-test or Mann–Whitney test (for continuous variables), or Chi-square test (for categorical variables) where applicable.

Lipid concentrations were log-transformed before the statistical analysis. The differences in plasma lipid levels between normal and no-reflow subjects were assessed from an independent Student’s *t*-test. The adjustment for multiple testing was made using a false discovery rate (FDR) approach using the Benjamini–Hochberg method [20]. The adjusted *p*-value (Q-value) cut-off was set to 0.2. All statistical analyses were conducted using the IBM Statistical Package for the Social Sciences (SPSS v24), and a *p*-value < 0.05 was considered statistically significant unless stated otherwise.

## 3. Results

### 3.1. Baseline Characteristics

Table 1 shows the study participants’ demographic, clinical, and laboratory characteristics stratified by CTFC. The subjects were divided into the normal flow and no-reflow groups according to the top CTFC tertile (≥30 frames/second) versus combined middle and bottom tertiles (CTFC < 30 frames/second). Patients in the no-reflow group were well balanced compared with the normal flow group for age, sex, BMI, and other risk factors (frequency of hypertension, diabetes mellitus, current smoking, dyslipidemia). Similarly, baseline lipid profile and concomitant medications (frequency of acetylsalicylic acid, angiotensin-converting enzyme inhibitor, β-blockers, statin) were comparable between the two groups. As expected, the no-reflow patients have a significantly longer time from symptom onset to PCI and a much higher post-PCI CTFC. The two groups also had comparable blood plasma levels of creatine kinase (peak CK) and cardiac troponin T (peak TnT), biomarkers of myocardial tissue injury.

### 3.2. Plasma Lipidome Alterations in the Setting of the NRP

**Figure 1:**
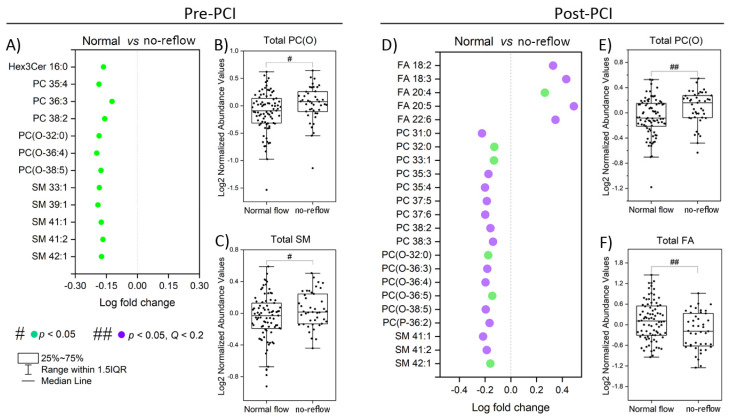
**Altered lipid species and classes during the coronary no-reflow phenomenon.** (**A**) The significantly altered lipid species between normal and no-reflow subjects at pre-PCI. (**B**,**C**) Boxplots of the abundance of lipid classes significantly differed between normal and no-reflow subjects at pre-PCI. (**D**) The significantly altered lipid species between normal and no-reflow subjects at post-PCI. (**E**,**F**) Boxplots of the abundance of lipid classes significantly differed between normal and no-reflow subjects at post-PCI. Green circles show lipid species with *p* < 0.05, and purple circles show lipid species with Q (corrected *p*) < 0.2 after independent Student’s *t*-test. # and ## represent statistical significance at *p* < 0.05 and Q < 0.2 after independent Student’s *t*-test.

Figure 1 shows the lipids significantly perturbed (*p* < 0.05) in no-reflow patients compared to normal flow patients at pre-PCI and post-PCI. Twelve lipid species were elevated considerably (*p* < 0.05) in no-reflow patients compared to normal flow patients at pre-PCI (Figure 1A). They mainly belong to phosphatidylcholine (PC), alkylphosphatidylcholine (PC(O)), and sphingomyelin (SM) classes. In addition, the total amount of two lipid classes, PC(O) and SM, was significantly higher (*p* < 0.05) in no-reflow patients than in normal flow patients (Figure 1B,C). Consistent with pre-PCI, 17 lipid species belonging to PC, PC(O), and SM lipid classes were also significantly elevated in no-reflow patients at post-PCI (Figure 1D). Surprisingly, fatty acids (FA) exhibited a reverse trend at post-PCI. The levels of five fatty acids (FA 18:2, FA 18:3, FA 20:4, FA 20:5, and FA 22:6), as well as the total fatty acid amount (Figure 1D,F), were significantly lower (*p* < 0.05) in no-reflow patients than normal flow patients at post-PCI, even after correction for multiple testing (Q < 0.2).

**Figure 2:**
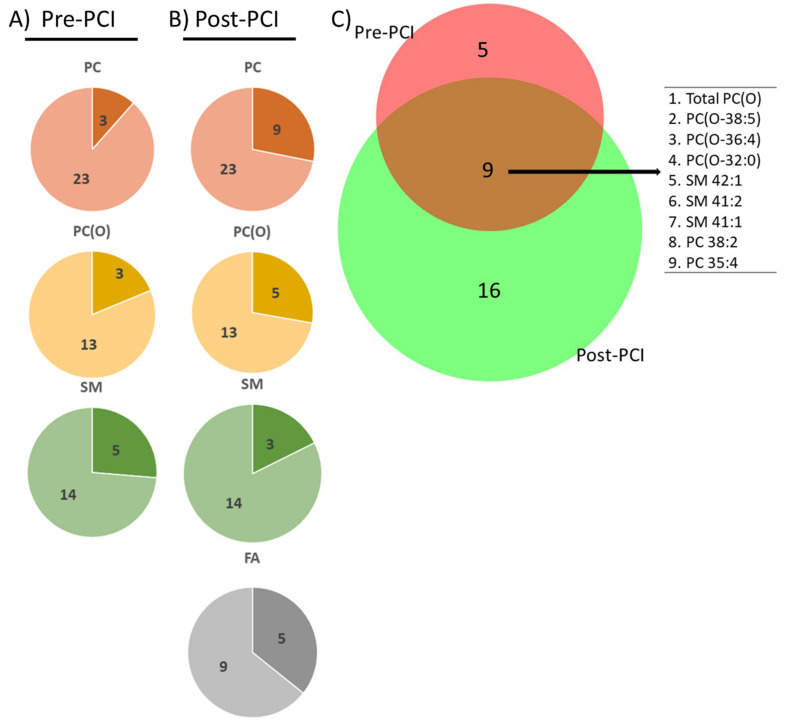
**Lipids at pre- and post-PCI**. The number of significantly altered lipid species (shown in a darker shade) at (**A**) pre-PCI and (**B**) post-PCI as parts of the corresponding whole lipid class. (**C**) Venn diagram shows the number of significantly altered lipid species (*p* < 0.05) unique or common between pre-PCI and post-PCI.

At pre-PCI and post-PCI, we detected 12 and 23 differentially expressed lipid species between normal and no-reflow patients, amounting to less than 1% of all measured lipid species (322 lipids). On the species level, 13% of PC, 23% of PC(O), and 35% of SM were altered at pre-PCI (Figure 2A), whereas 39% of PC, 38% of PC(O), 21% of SM, and 55% of FA were altered at post-PCI (Figure 2B). Notably, eight lipid species (excluding total PC (O)), including two PC species (PC 35:4 and PC 38:2), three SM species (SM 41:1, SM 41:2, and SM 42:1), and three PC(O) species (PC(O-32:0), PC(O-36:4), and PC(O-38:5)) were significantly altered (*p* < 0.05) between normal and no-reflow patients at both pre-PCI and post-PCI time intervals (Figure 2C). The total PC(O) amount is also significantly altered at pre-PCI and post-PCI (Figure 2C).

**Figure 3:**
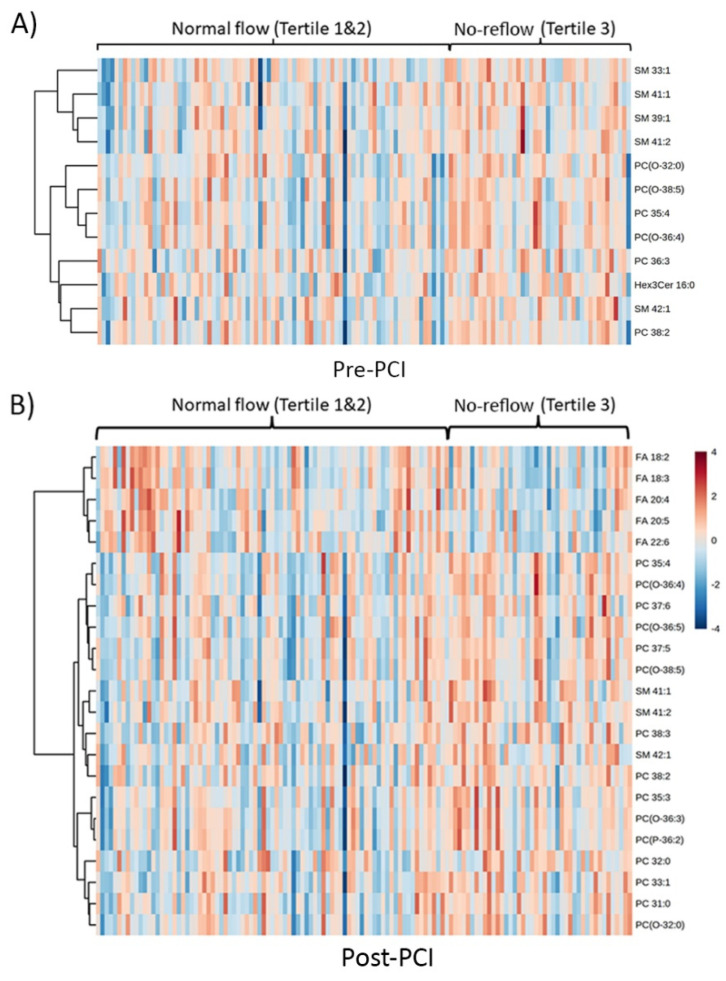
**Clustered heatmap of significantly altered lipid species**. A hierarchically clustered heat map representing significantly altered lipid species (*p* < 0.05) between normal and no-reflow subjects at (**A**) pre-PCI and (**B**) at post-PCI. Data were normalized, log-transformed, and auto scaled.

A heatmap of statistically significant lipids (Figure 3) was constructed to visualize the intensity variations between the normal and no-reflow patients at pre-PCI and post-PCI. The heatmap was generated (using default parameters) using the web-based tool MetaboAnalyst (www.metaboanalyst.ca), accessed on 1 October 2021. Except for fatty acids, the levels of all other lipids were generally higher in the no-reflow group than in the normal flow group during both periods (Figure 3A,B). Interestingly, the fatty acid profile was distinct from the rest of the lipids at post-PCI. In the heatmap, related lipids are grouped by hierarchical clustering. Notably, all the fatty acids exhibited a tight clustering post-PCI (Figure 3B), indicating their similarity in action after reperfusion.

### 3.3. Association of Clinical Parameters of No-Reflow with Circulating Lipids

**Figure 4:**
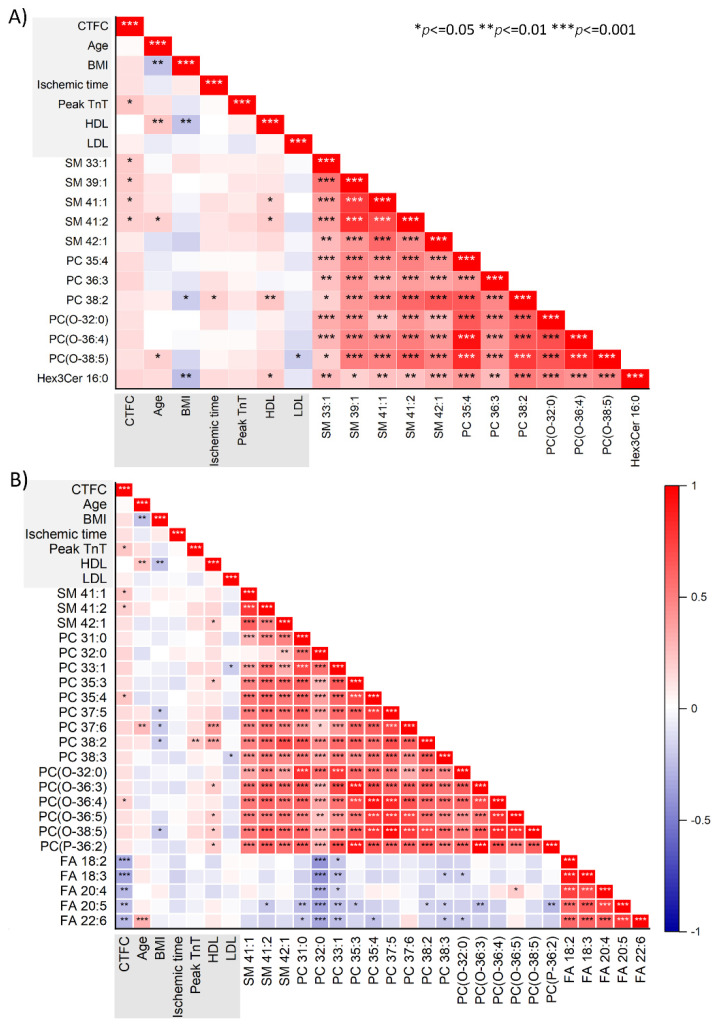
**Correlation between lipid species and clinical parameters.** The correlation plots depict the strength of the relationship (Pearson correlation) between significantly altered lipid species (*p* < 0.05) and clinical parameters at (**A**) pre-PCI and (**B**) post-PCI. Positive correlations are displayed in red and negative correlations in blue. *, **, *** represents the significant level at *p* < 0.05, 0.01, 0.001, respectively.

The results of correlation analysis (Pearson correlation) between circulating lipids and the clinical factors associated with no-reflow were illustrated as a correlation matrix (Figure 4). The correlation analysis displayed two clusters, (1) a cluster with all no-reflow-associated PC, PC(O), and SM species and (2) a cluster with fatty acids alone. The PC, PC(O), and SM species were positively associated (*p* < 0.001) with each other and among themselves at pre-PCI and post-PCI. The fatty acids were positively associated (*p* < 0.001) with each other at post-PCI but not with other no-reflow-associated lipids. The correlation analysis also revealed a significant (*p* < 0.05) positive correlation between CTFC and two SM species, namely SM 41:1 and 41:2 at pre-PCI and post-PCI. The post-PCI levels of all fatty acids exhibited inverse correlations with CTFC (*p* < 0.01).

### 3.4. Temporal Perturbations in Select Lipid Species

**Figure 5:**
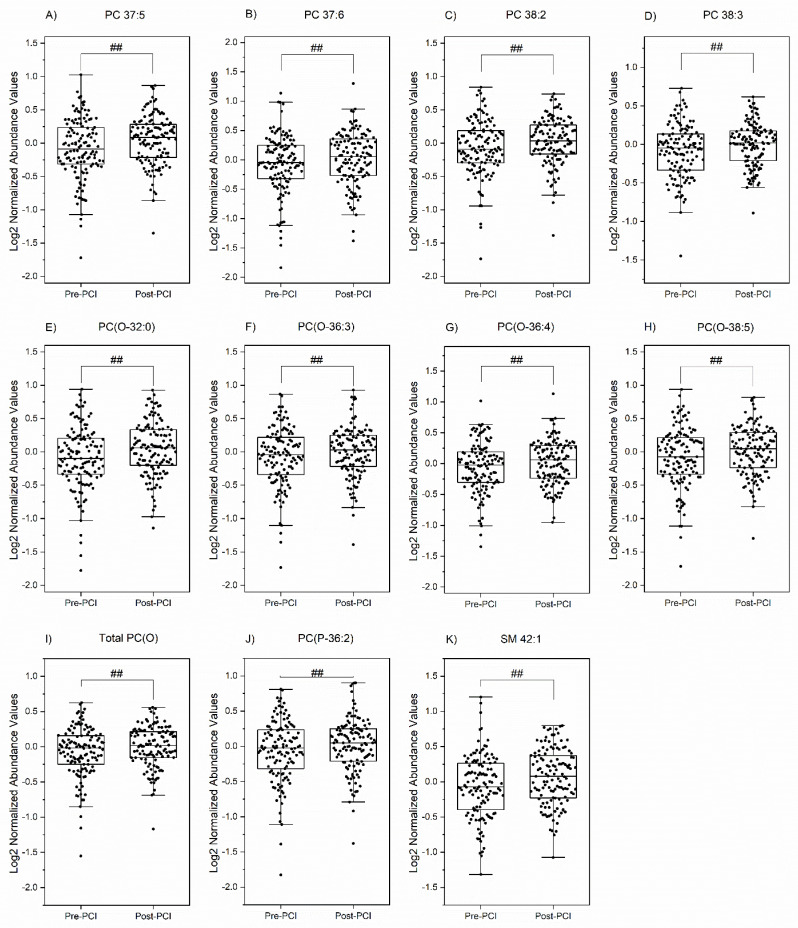
**Time-dependent changes in lipid species.** The box plots show the significantly altered lipid species (*p* < 0.05) in time (between pre-PCI and post-PCI). A paired *t*-test was run on the list of 26 lipids and 4 lipid classes (significantly different between normal and no-reflow patients) to determine whether there was a statistically significant difference in time. *##* represents the significant level at *Q* (corrected *p*) < 0.05.

We next investigated the changes over time in select lipid species, which were predicted to play essential roles in the setting of NRP. Of the total 26 lipids that were differentially abundant between normal and no-reflow patients based on CTFC, 10 lipids were also significantly different (Q < 0.05) between pre- and post-PCI (Figure 5). Interestingly, these included four PC species (PC 37:5, PC 37:6, PC 38:2, and PC 38:3) and four PC(O) species (PC(O-32:0), PC(O-36:3), PC(O-36:4), and PC(O-38:5)). The total PC(O) amount is also significantly altered between pre- and post-PCI.

### 3.5. Association of Lipids with ST-Segment Resolution (STR)

**Figure 6:**
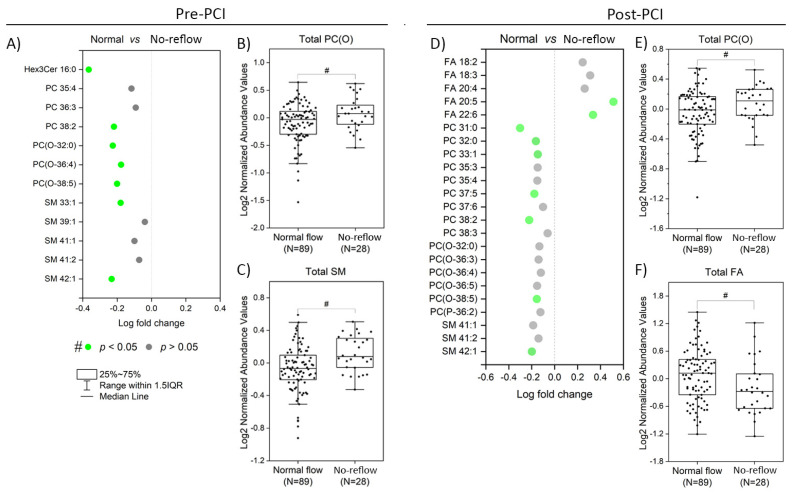
**Altered lipid species and classes based on ST segment resolution** (**A**) The significantly altered lipid species between normal and no-reflow subjects (ST resolution < 50%) at pre-PCI. (**B**,**C**) Boxplots of the abundance of lipid classes significantly differed between normal and no-reflow subjects (ST resolution < 50%) at pre-PCI. (**D**) The significantly altered lipid species between normal and no-reflow subjects (ST resolution < 50%) at post-PCI. (**E**,**F**) Boxplots of the abundance of lipid classes significantly differed between normal and no-reflow subjects (ST resolution < 50%) at post-PCI. Green circles show lipid species with *p* < 0.05, and grey circles show lipid species with *p* > 0.05 after independent Student’s *t*-test. *#* represent statistical significance at *p* < 0.05 after independent Student’s *t*-test.

ST-segment monitoring in serial ECG is another simple means of assessing myocardial perfusion following PCI [21]. ST-segment resolution (STR) is increasingly used in clinical research as a marker of no-reflow. We then explored whether the lipids associated with CTFC were also associated with STR. Based on STR, we again categorized the patients into two groups, normal and no-reflow. Those with STR < 50% were classified as no-flow patients, and those with STR > 50% were grouped into normal-flow patients. Based on STR classification, 7 of the 12 differential lipids identified via CTFC classification at pre-PCI significantly differed between normal and no-reflow patients (Figure 6). Similarly, 9 out of 23 differential lipids were common in post-PCI CTFC and STR classifications. Interestingly, all the differential lipids exhibited the same trend regarding increment or decrement of their amounts in no-reflow and normal flow patients with both groupings.

### 3.6. Correlations between Plasma Cytokine Levels and No-Reflow-Associated Lipids

**Figure 7:**
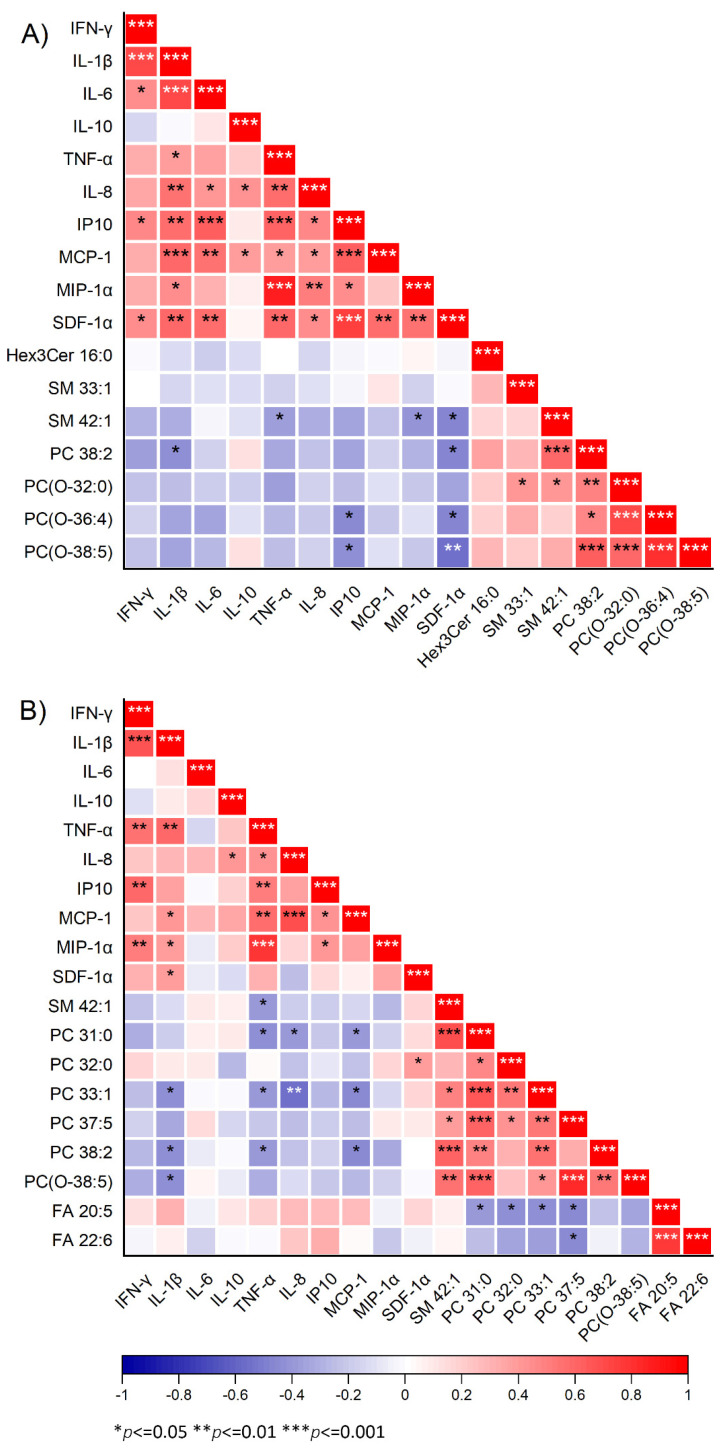
**Correlation of cytokines with lipid species.** The plot shows the association of significantly altered lipids based on both corrected TIMI frame count (CTFC) and ST-segment resolution with 10 cytokines at pre-PCI (**A**) and post-PCI (**B**) for 28 subjects. The red color symbolizes positive correlation, and the blue color symbolizes negative correlation. *, **, *** represents the significant level at *p* < 0.05, 0.01, 0.001, respectively.

Inflammation is an essential component of myocardial ischemia/reperfusion (IR) injury that underlies no-reflow development [22]. Therefore, we decided to assess the interactions between lipid metabolism and immune response in our cohort (in a subset of 28 STEMI patients). To this end, we choose a panel of 10 cytokines and chemokines associated with inflammatory responses during IR injury. A correlation matrix is displayed in Figure 7. Two chemokines, namely stromal cell-derived factor 1α (SDF-1α) and interferon-γ-induced protein-10 (IP-10), were correlated inversely (*p* < 0.05) with two ether-linked PCs, namely PC(O-36:4), and PC(O-38:5) at pre-PCI (Figure 7A). Our data also showed inverse correlation (*p* < 0.05) between two PC species, namely PC 33:1 and PC 38:2, and circulating levels of three pro-inflammatory cytokines, namely interleukin 1 beta (IL-1β), tumor necrosis factor-alpha (TNF-α), and monocyte chemoattractant protein-1(MCP-1) at post-PCI (Figure 7B). SM 42:1 exhibited an inverse correlation (*p* < 0.05) with TNF-α at pre-PCI and post-PCI. Notably, none of the no-reflow-associated lipids were associated with the anti-inflammatory cytokine IL-10 at both intervals.

## 4. Discussion

Our study describes the detailed changes in human plasma lipidome in the setting of NRP in patients undergoing primary PCI. Our results revealed that STEMI patients who develop no-reflow have a distinct lipidomic signature compared to those with normal flow. We found that (1) levels of specific species of phosphatidylcholine (PC), alkylphosphatidylcholine (PC(O)), and sphingomyelin (SM) were significantly elevated (*p* < 0.05) in no-reflow patients; (2) the levels of circulating fatty acids were markedly lower (*p* < 0.05) in the no-reflow group than the normal flow group at post-PCI; (3) no-reflow associated lipids were significantly perturbed (*p* < 0.05) before and after reperfusion; and 4) specific SM species exhibited a positive association with CTFC and an inverse association with TNF-α, a pro-inflammatory cytokine.

The levels of PC (O-32:0), PC(O-36:4), and PC(O-38:5), as well as total PC(O) levels, were significantly higher (*p* < 0.05) in the no-reflow group compared to the normal flow group. This persists even after correction for multiple comparisons at post-PCI (Q < 0.2). PC(O) is an ether-linked phospholipid in which an ether bond attaches the hydrocarbon chain at the *sn-1* position of the glycerol backbone instead of the more common ester bond [23]. In addition to their structural roles (e.g., lipid packing), ether-linked PCs exert diverse functions ranging from regulating cell differentiation to lipid signaling and inflammation [23,24]. There is also evidence that ether lipids protect against oxidative damage by functioning as endogenous antioxidants [23]. This is particularly interesting in the setting of NRP, where oxidative stress plays a key role [25]. No-reflow is a process that starts with lethal ischemia and increases during reperfusion [3,26]. Within the first few minutes of reperfusion, oxygen-free radicals are produced in excess through multiple pathways, leading to additional myocardial damage, known as myocardial ischemia/reperfusion injury [27,28]. Our findings show a marked increase in the levels of ether-linked PCs in the reperfused state (post-PCI) compared to the ischemic state (pre-PCI). In the current study, the paradoxical increase in circulating levels of ether-linked PCs in no-reflow patients and at reperfused state might be an antioxidant response to increased oxidative stress under these conditions.

Our analysis also revealed that the elevated phospholipids (PC, PC(O), and SM) levels in no-reflow patients at post-PCI are accompanied by a relative reduction in free fatty acid levels, including arachidonic acid (AA; C20:4) and docosahexaenoic acid (DHA; C22:6). These fatty acids were also significantly (negative) correlated with CTFC at post-PCI. Ether-linked PCs, such as those identified in the current study (36:4, 36:5, 38:5) are highly unsaturated and comprise AA as the major fatty acid moiety at the *sn-2* position. Polyunsaturated fatty acids (PUFAs) can be released due to increased phospholipase A2 (PLA2) activity [29]. AA can then be enzymatically oxidized to form bioactive signaling lipids known as eicosanoids, which have recently been implicated in many human diseases, including diabetes and cardiovascular disease (CVD) [30]. They also serve essential functions in cardiovascular homeostasis, such as regulating vascular tone [31]. Since AA is found almost entirely in phospholipids, our current study’s observed decrease in arachidonate suggests reduced PLA2 activity in no-reflow patients after coronary intervention. However, it is yet to be seen whether this deficit of arachidonic acid in no-reflow patients leads to reduced biotransformation of arachidonate to AA-derived eicosanoids.

We found that plasma sphingomyelin levels (SM 41:1, SM 41:2, and SM 42:1) were consistently elevated in the no-reflow group at pre-PCI and post-PCI. Of these, the levels of SM 41:1 and SM 41:2 were also positively associated with the levels of CTFC at pre-PCI and post-PCI. This data agrees with two recent reports, which have demonstrated that sphingolipids are increased in atherosclerotic plaques compared with normal arteries and that the major source is plasma lipoproteins [32,33,34]. A lipidomic analysis on the plaques isolated from hypercholesterolemic rabbits showed that SM was present in relatively higher amounts within the atherosclerotic lesions [32]. Consistent with this is the observation that levels of several sphingolipids, including SM, are elevated in symptomatic human atherosclerotic plaques [33]. In another study involving human patients, Tanaka et al. [35] demonstrated that lipid content from culprit plaques is an independent predictor of no-reflow. These data suggest that elevated plasma SM levels in no-reflow patients likely reflect the higher SM content in the lipid-rich atherosclerotic plaque. The physical destruction of this plaque during PCI may induce an outflow of soluble factors, including lipids from the culprit lesion, into the coronary circulation [35] and may trigger no-reflow.

Inflammatory indices, such as blood eosinophil count [36] and neutrophil-to-lymphocyte-ratio [37], are useful for predicting no-reflow. However, our data failed to show any association between plasma levels of inflammatory cytokines and coronary no-reflow, as assessed by CTFC. This finding is in line with a previous interventional study [38], which showed that baseline systemic inflammation, as assessed by serum CRP, is not associated with NRP. In general, the correlation between levels of cytokines and no-reflow-associated lipids was inconsistent at pre-PCI and post-PCI. Strikingly, the levels of TNF-α were consistently significantly correlated (*p* < 0.05) with levels of SM 42:1 at pre-PCI and post-PCI. TNF-α is a key regulator of MI and IR injury [39] through several signaling pathways, including the production of reactive oxygen species (ROS), synthesis of nitric oxide (NO), or activating sphingomyelinase [39]. TNF-α also participates in regulating lipid metabolism, including sphingolipid metabolism [39,40]. There is evidence that the action TNF-α, in part, is mediated by the SM signal transduction pathway [41,42]. TNF-α is a known agonist of sphingomyelinase, which hydrolyze SM to generate ceramides [43]. This conversion is fast and depends on the cell types involved. The association between SM 42:1 and TNF-α suggests the regulation of immune responses by sphingomyelins during no-reflow in STEMI patients after PCI.

The NRP is associated with the worst outcome, especially in patients with STEMI undergoing PCI [44]. However, the mechanism of this phenomenon remains elusive. Our analysis showed that lipid alterations are associated with NRP after PCI. Our data also showed that no-reflow-associated lipids biomarkers (PC, PC(O), SM, and fatty acids) could help us to identify patients at high risk for no-reflow in the catheterization laboratory. These results have the potential for therapeutic intervention in the setting of no-reflow in STEMI patients.

## 5. Limitations

Firstly, though the present investigation demonstrated novel lipid signatures associated with no-reflow, the molecular basis of these findings remains elusive. Translating these results (body fluid readouts) to target tissues (here, the myocardium) is challenging. One way to address this issue is by combining animal models of no-reflow with omics platforms, such as proteomics, metabolomics, and lipidomics. This approach will help to unravel specific mechanisms and also help to establish the causal link between circulating lipids and NRP.

Secondly, this single-center study lacks a replication cohort. Therefore, extrapolation of the current findings to other populations is challenging. One way to overcome this is by conducting independent metabolomics studies in diverse STEMI cohorts (multi-centric) to evaluate our results. Our lipidomics platform does not resolve fatty acyl chains’ sn-position (composition and position of fatty acyl chain) on the glycerol backbone. Therefore, there is the likelihood that identified lipid markers are not unique molecules but represent a mixture of molecular isomers (e.g., SM 42:1). The determination of acyl chain composition requires extensive chromatographic or ion-mobility separations or derivatization methods. Future studies should be directed to address this issue as well.

## 6. Conclusions

In conclusion, by simultaneously quantifying over 300 lipid species in plasma, we identified lipid species and lipid pathways dysregulated in a clinical setting of no-reflow. This report is the first large scale lipidomic analysis of patients undergoing reperfusion therapy stratified by coronary no reflow. Our analysis showed that lipid abnormalities, mainly involving PC, PC(O), SM, and fatty acid species, were associated with no-reflow development. Our results highlight the value of lipidomics in identifying new biomarkers of disease risk and offer a better understanding of the underlying mechanisms involved in disease pathogenesis.

## Figures and Tables

**Table 1:** Baseline characteristics of the study participants.

	Normal Flow	No-Reflow Flow	
	Tertile-1&2 (*n* = 83)	Tertile-3 (*n* = 43)	*p* Value
Age (years)	62 (51, 74)	62 (58, 69)	0.75
Male sex (%)	61 (73.5)	36 (83.7)	0.196
LVEF (%)	60 (44.7, 68.2)	58.5 (43, 66.2)	0.431
Body mass index (kg/m^2^)	27.4 (23.5, 31.2)	28.8 (26.3, 32.8)	0.148
**Comorbidity (%)**			
Hypertension	29 (34.9)	21 (48.8)	0.131
Diabetes mellitus	17 (20.5)	7 (16.3)	0.569
Current smoker	29 (34.9)	10 (23.3)	0.179
Dyslipidemia	43 (51.8)	17 (39.5)	0.191
Hx of CAD	17 (20.5)	6 (14.0)	0.368
**Laboratory data**			
Triglyceride (mmol/L)	1.4 (1, 2.3)	1.2 (1, 1.9)	0.421
Cholesterol (mmol/L)	4.58 ± 1.15	4.62 ± 1.16	0.889
HDL cholesterol (mmol/L)	1.1 (0.9, 1.4)	1.0 (0.85, 1.3)	0.589
LDL cholesterol (mmol/L)	2.6 (1.7, 3.3)	2.8 (2.1, 3.5)	0.244
Creatinine (mmol/L)	85.5 (72, 102.5)	94 (78, 114)	0.113
**Medications at baseline (%)**			
ASA	21 (25.3)	9 (20.9)	0.585
ACEI/ARB	17 (20.5)	11 (25.6)	0.514
Beta blocker	10 (12)	3 (7)	0.375
Statin	23 (27.7)	7 (16.3)	0.153
**Additional parameters**			
CTFC (frames)	20 (16, 24)	40 (35.3, 49.4)	<0.0001
Ischemic time (min)	163 (105, 244)	247 (125, 479)	0.027
Peak CK (Units/L)	949 (373, 2591)	1224 (676, 3092)	0.213
Peak TnT (ng/L)	2259 (882, 5791)	3575 (1229, 8689)	0.093
**Culprit vessel (%)**			
LAD Infarct (%)	35 (42.2)	22 (51.2)	0.336
RCA Infarct (%)	43 (51.8)	12 (27.9)	0.01
Circumflex Infarct (%)	7 (8.4)	10 (23.3)	0.021

Values are reported as mean ± standard deviation (SD), median (25th, 75th percentiles), or count (percentage) as applicable. The Chi-square test was used for categorical variables, while Student’s *t*-test or Mann–Whitney U test was used for continuous variables to assess for statistical significance across sample groups as applicable based on data distribution. Abbreviations: CTFC = corrected TIMI frame count; LVEF  =  left ventricular ejection fraction; Hx of CAD  =  history of coronary artery disease; HDL = high-density lipoprotein; LDL  =  low-density lipoprotein; ASA  =  Acetylsalicylic acid; ACEI  =  Angiotensin-converting enzyme (ACE) inhibitors; ARB  =  Angiotensin II receptor blockers; CK  =  Creatine kinase; TnT  =  troponin T; LAD  =  Left anterior descending coronary artery; RCA  =  Right coronary artery.

## Data Availability

The data presented in this study are available in the publicly available EMBL-EBI MetaboLights database with the identifier MTBLS3839.

## Supplementary Materials

The following supporting information can be downloaded at: https://www.mdpi.com/article/10.3390/metabo13010079/s1, Video S1: normal flow coronary angiogram; Video S2: no-reflow coronary angiogram.
